# Five-year structural changes in severe high myopia: the role of choroidal and retinal thickness

**DOI:** 10.1186/s12886-026-05076-1

**Published:** 2026-07-28

**Authors:** Jay Jiyong Kwak, Seung Jae Lee, Christopher Seungkyu Lee, Suk Ho Byeon, Sung Soo Kim

**Affiliations:** 1 Institute of Vision Research, Department of Ophthalmology, Severance Eye Hospital, Yonsei University College of Medicine, Seoul, Republic of Korea; 2 Yonsei Institute for Digital Health, Yonsei University, Seoul, Republic of Korea

**Keywords:** High myopia, Choroidal thickness, Retinal thickness, Choroidal-to-retinal thickness ratio

## Abstract

**Background:**

High myopia is associated with vision-threatening complications and progressive structural changes of the posterior segment. While choroidal thickness is a sensitive marker of myopia-related axial elongation, combining choroidal and retinal thickness into a choroidal-to-retinal thickness (CR) ratio may better capture clinically relevant chorioretinal alterations. This study aimed to evaluate 5-year changes in choroidal thickness, retinal thickness, and the CR ratio and their associations with axial elongation in severe high myopia.

**Methods:**

This retrospective study included 62 eyes from 37 patients with high myopia (axial length ≥ 26.0 mm; spherical equivalent ≤ -6.0 D), excluding eyes with pathologic myopia, foveoschisis, or macular hole; eyes with posterior staphyloma were included. Optical coherence tomography was used to measure subfoveal and perifoveal choroidal thickness and retinal thickness. The CR ratio was analysed in relation to age, axial length, axial elongation rate, and posterior staphyloma. Multivariable regression identified predictors of axial elongation and staphyloma.

**Results:**

The cohort represented a severely myopic spectrum (mean axial length 30.80 ± 1.96 mm; mean spherical equivalent − 17.3 ± 5.10 D; mean age 46.5 ± 16.2 years), hereafter referred to as severe high myopia. Axial length increased from 30.80 to 31.17 mm over 5 years (*P* < 0.001), while subfoveal choroidal thickness (67.76 to 58.63 μm; *P* = 0.005), foveal retinal thickness (203.34 to 195.42 μm; *P* = 0.042), and the subfoveal CR ratio (0.33 to 0.30; *P* = 0.036) decreased. The baseline CR ratio inversely correlated with age, axial length, and refractive error (R² = 0.15–0.48, all *P* < 0.01). A higher subfoveal CR ratio was independently associated with a reduced risk of rapid axial elongation (OR 0.032; 95% CI, 0.003–0.395; *P* = 0.007), along with older age and lower baseline foveal retinal thickness. Axial length was the strongest predictor of staphyloma (OR 10.367; *P* = 0.005).

**Conclusions:**

Subfoveal choroidal thickness declined progressively with age and axial elongation, serving as a sensitive marker of myopic progression. A higher subfoveal CR ratio independently predicted a lower risk of rapid axial elongation, suggesting its potential as a prognostic biomarker in severe high myopia.

**Supplementary Information:**

The online version contains supplementary material available at https://doi.org/10.1186/s12886-026-05076-1.

## Background

 Myopia is a global public health issue with rapidly increasing prevalence and substantial clinical and socioeconomic burdens [1]. Individuals with high myopia are at elevated risk for vision-threatening complications, including myopic maculopathy, retinal detachment, glaucoma, macular degeneration, and cataracts. Although the pathogenesis of myopia remains to be fully understood, accumulating evidence suggests that the choroid plays a central role [2]. Eyes can respond to optical defocus within minutes by modulating choroidal thickness (CT) and axial elongation [3–5]. Under hyperopic defocus (where the image is focused behind the retina), the choroid undergoes rapid thinning as part of the eye’s compensatory response. These choroidal changes are thought to influence scleral remodelling and contribute to long-term axial elongation [6, 7].

Retinal thickness (RT) shows a weaker correlation with axial length and refractive error than CT, and its association with visual acuity is less consistent in highly myopic eyes [8, 9]. Although some studies report increased foveal thickness in myopic eyes, parafoveal and perifoveal RTs often decline due to axial elongation [10, 11]. Consequently, CT has previously been considered a more sensitive structural biomarker. However, retinal thinning, particularly within the myoid and ellipsoid zones, has been associated with reduced visual acuity [12]. Additionally, photoreceptor layer thinning and decreased outer retinal thickness have been correlated with poorer vision and light sensitivity, even in highly myopic eyes without overt maculopathy [13, 14]. Therefore, we hypothesize that the novel choroidal-to-retinal thickness (CR) ratio may better reflect chorioretinal changes than either metric alone. In this 5-year longitudinal study of eyes with severe high myopia, we aimed to: (1) analyse CR ratio correlations with age, axial length, refractive error, and visual acuity; (2) evaluate long-term changes in CR ratio across different age and axial elongation subgroups; and (3) assess structural risk factors associated with axial elongation and posterior staphyloma, key contributors to myopic maculopathy [15–18].

## Methods

We retrospectively reviewed the medical records of consecutive patients with high myopia who visited Severance Eye Hospital, Seoul, South Korea, between January 2014 and June 2016. This study was performed in accordance with the ethical standards laid down in the 1964 Declaration of Helsinki and was approved by the Institutional Review Board of the Yonsei University College of Medicine (IRB no. 4-2023-0189). The requirement for informed consent was waived. High myopia was defined as an axial length ≥ 26 mm and a spherical equivalent ≤ -6.00 D. Only patients with a minimum of 5 years of follow-up and at least one clinic visit per year were included. Patients were excluded if they had any signs of pathologic myopia, including diffuse chorioretinal atrophy, patchy chorioretinal atrophy, macular atrophy, lacquer cracks, myopic choroidal neovascularization, or Fuchs spots according to the International Meta-Analysis for pathologic myopia (META-PM) classification, as well as foveoschisis or macular hole. Eyes with posterior staphyloma were not excluded. Patients with a history of vitrectomy or any other procedure involving the macula were also excluded. Of the 62 included eyes, 24 had undergone cataract surgery or corneal refractive surgery. These eyes were excluded from analyses involving spherical equivalent. However, they were retained for axial length analyses, as both cataract surgery and laser refractive surgery have been shown to have clinically negligible effects on long-term axial length measurements [19–21].

Data were collected on patient demographics, best-corrected visual acuity (BCVA, logMAR), refractive error, and axial length. Axial length was measured using the IOL Master 500 (Carl Zeiss Meditec, Jena, Germany) through 2018 and the IOL Master 700 (Carl Zeiss Meditec, Jena, Germany) thereafter, and spherical equivalent was obtained using an auto kerato-refractometer (Topcon KR-1, Tokyo, Japan). All measurements were performed under pharmacologic pupil dilation. All patients underwent dilated fundus examination and ultrawide-angle fundus photography (Optos, Dunfermline, UK). Spectral-domain optical coherence tomography (OCT) was performed after pharmacologic pupil dilation at each routine visit using the Spectralis OCT system (Heidelberg Engineering, Heidelberg, Germany) and scans were analysed with Heidelberg Eye Explorer (HEYEX) software. High-resolution horizontal line scans were acquired using a 30° × 25° raster protocol (9.3 × 7.7 mm) composed of 61 B-scans (768 A-scans per B-scan) with 129-µm inter-scan spacing and real-time averaging of 29 frames. Scans were carefully centred on the foveal pit, identified by the deepest foveal contour and a distinct foveal reflex. Foveal RT and subfoveal CT were manually measured using the distance measurement tool included in the HEYEX software for Spectralis OCT at baseline, and at 1, 3, and at 5 years. For foveal RT, the distance from the foveal centre to Bruch’s membrane was measured. For subfoveal CT, distance from Bruch’s membrane to choroid-scleral junction was measured. Perifoveal RT and CT was measured in the same manner at a point located 1,000 μm temporal to the foveal centre. The temporal side was chosen for choroidal measurements, as nasal CT is generally thinner and more variable in highly myopic eyes [22, 23]. All measurements were independently performed by two examiners (JJK and SJL) and averaged. To calculate interobserver variability, intraclass correlation coefficient was analyzed. CR ratio was calculated at both the subfoveal and perifoveal locations. Posterior staphyloma was defined as a localised outpouching of the ocular wall with a smaller radius of curvature than that of adjacent areas, as confirmed by OCT cross-Sects [24, 25]. Only eyes with narrow macular, wide macular, or peripapillary type staphylomas involving the foveal or perifoveal regions were included. Axial elongation rate was defined as the change in axial length (mm/year) between baseline and year 5. Eyes were stratified into rapid (≥ 0.1 mm/year) and moderate (< 0.1 mm/year) elongation groups [17]. The primary outcome was the change in subfoveal and perifoveal CR ratios.

To account for the non-independence of fellow eyes from the same patient, all primary analyses were performed using generalized estimating equations (GEE) with patient as the clustering unit. An exchangeable working correlation structure was applied for all continuous outcomes, including longitudinal changes in axial length, choroidal thickness, retinal thickness, and the choroidal-to-retinal thickness ratio, as well as for univariable correlation and multivariable linear regression analyses at baseline. For longitudinal analyses, time was modelled as a categorical variable with baseline as the reference. For logistic regression analyses, including predictors of rapid axial elongation and posterior staphyloma, an independence working correlation structure was used to ensure model convergence. A Gaussian family was specified for continuous outcomes and a binomial family with logit link for binary outcomes. GEE analyses were performed using Python 3.12 (statsmodels library, version 0.14.6). Descriptive statistics and non-GEE analyses, including the intraclass correlation coefficient, were performed using SPSS version 28.0 (IBM Corp., Armonk, NY, USA).

## Results

### Baseline correlations of choroidal and retinal thickness with clinical parameters

**Fig. 1:**
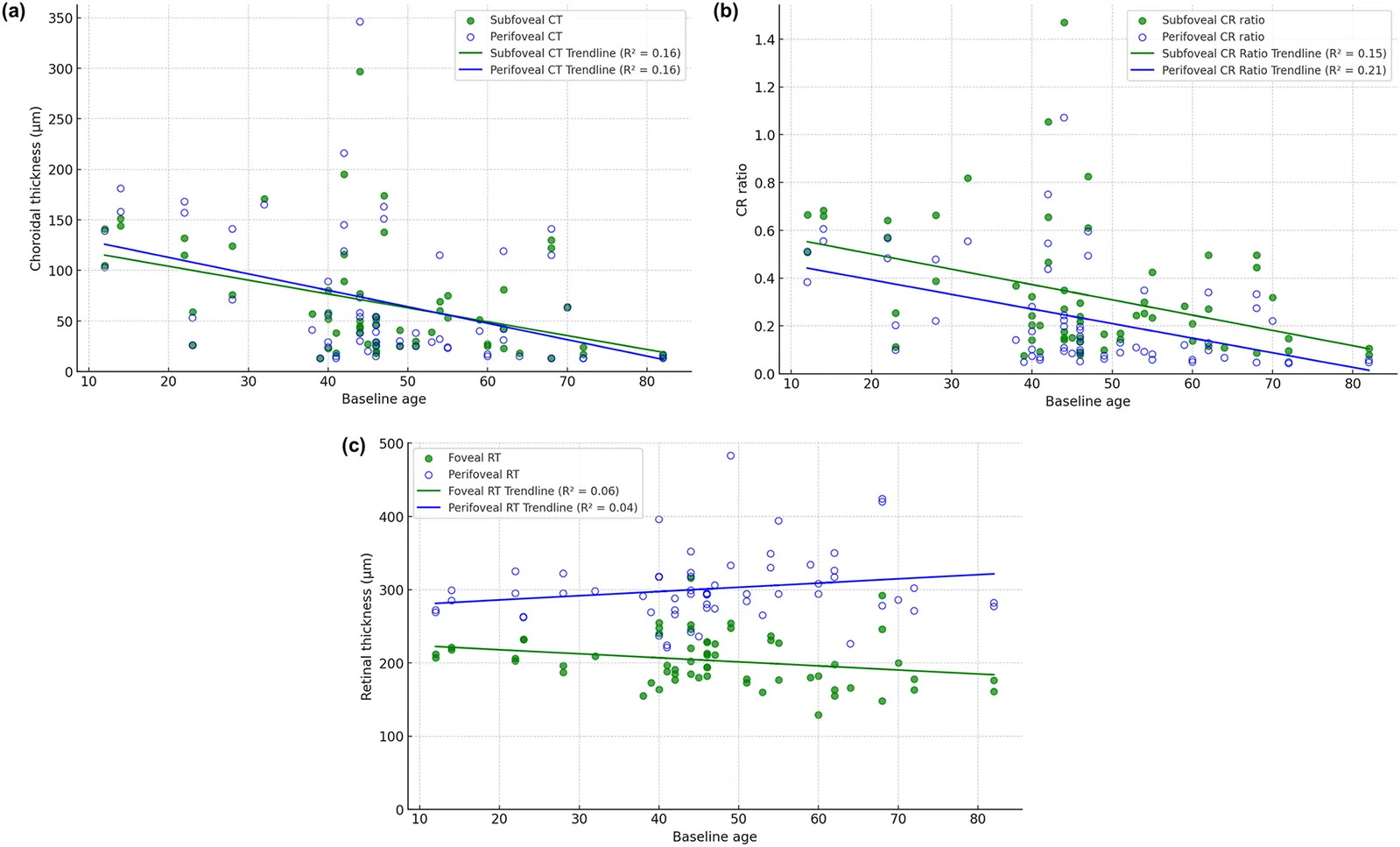
Retinal and choroidal thickness in relation to age. Scatter plots of (**a**) choroidal thickness, (**b**) choroidal-to-retinal thickness ratio, and (**c**) retinal thickness. Subfoveal/foveal values (green); perifoveal values (blue). CR ratio, choroidal-to-retinal thickness ratio; CT, choroidal thickness; RT, retinal thickness

**Fig. 2:**
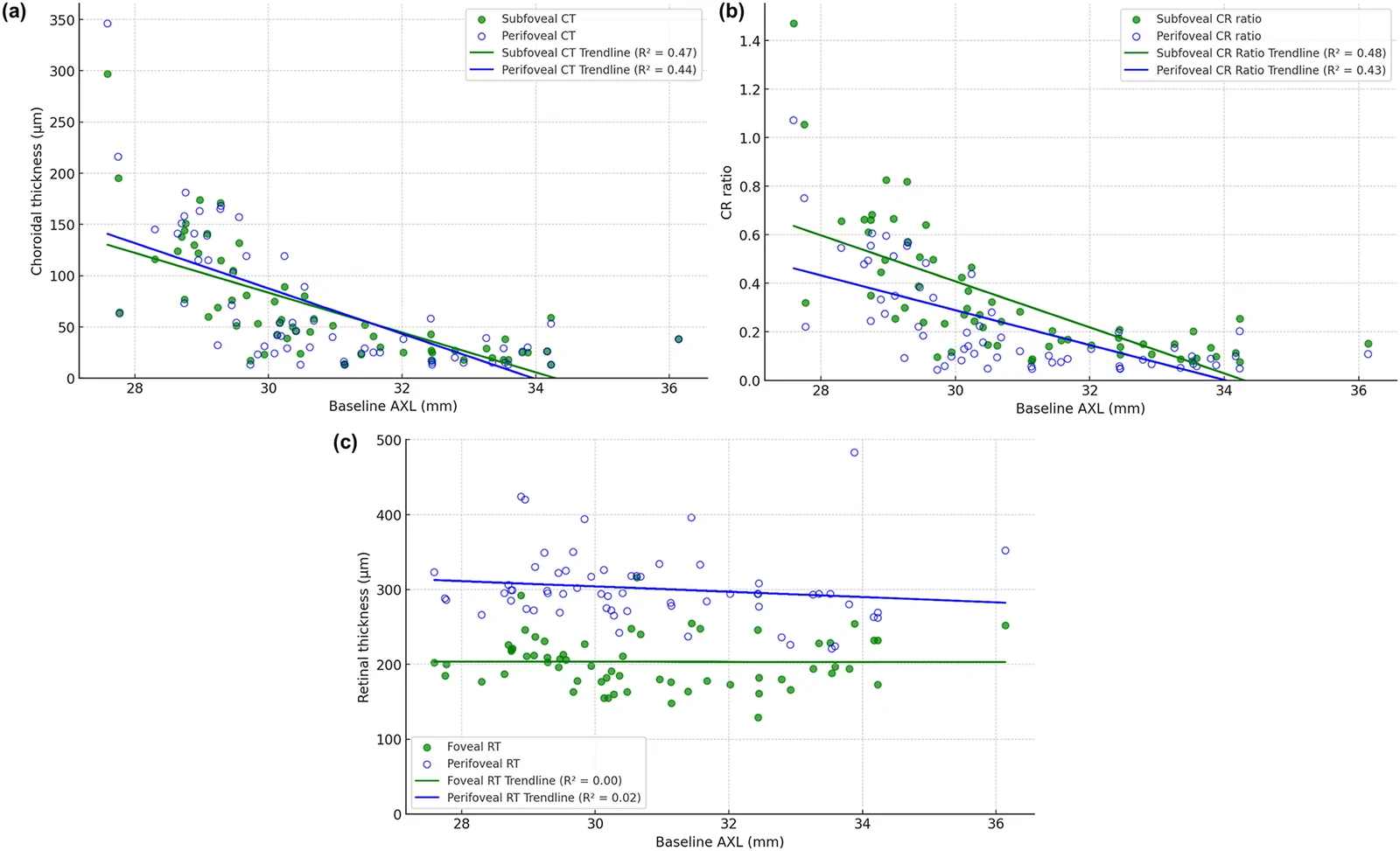
Retinal and choroidal thickness in relation to baseline axial length. Scatter plots of (**a**) choroidal thickness, (**b**) choroidal-to-retinal thickness ratio, and (**c**) retinal thickness. AXL, axial length; CR ratio, choroidal-to-retinal thickness ratio; CT, choroidal thickness; RT, retinal thickness

Sixty-two eyes from 37 patients (78.4% female) with a mean age of 46.5 ± 16.2 years were included (Table 1). Interobserver reliability for choroidal and retinal thickness measurements was high, with an intraclass correlation coefficient (ICC) greater than 0.900 (*P* < 0.01). The mean baseline refractive error and axial length were − 17.3 ± 5.10 D and 30.80 ± 1.96 mm, respectively, substantially exceeding the conventional high myopia thresholds and falling within the range previously classified as severe myopia (spherical equivalent ≤ − 14 D) [26]. Throughout this manuscript, we therefore refer to this cohort as severe high myopia, reflecting the extreme degree of axial elongation and refractive error rather than a separate diagnostic category. The mean axial elongation rate over 5 years was 0.07 ± 0.05 mm/year. At baseline, both subfoveal and perifoveal CT and CR ratios showed significant negative correlations with age (R² = 0.15–0.21), axial length (R² = 0.43–0.48), refractive error (R² = 0.44–0.45), and logMAR BCVA (R² = 0.13–0.15) (all *P* < 0.05; Figs. 1a-b and 2a-b; Additional files 1, 2). In contrast, RT showed only weak or no association with these parameters (R² = 0.001–0.08, all *P* > 0.05 except foveal RT versus age: R² = 0.06, *P* = 0.048) (Figs. 1c and 2c).

In multivariable GEE analysis, axial length, age, and axial elongation rate were independently associated with the subfoveal CR ratio (all *P* < 0.05, Additional file 3), while axial length, age, and female sex were significant predictors for the perifoveal CR ratio (all *P* < 0.050).

**Table 1:** Baseline demographic and clinical characteristics

	mean ± SD
Total no. of eyes, *n*	62
Age (years)	46.5 ± 16.2
Female sex, n (%)	29 (78.4)
Baseline BCVA (logMAR)	0.20 ± 0.24
Refractive error (D)^*^	−17.3 ± 5.10
Baseline axial length (mm)	30.80 ± 1.96
Axial elongation rate/year (mm/year)	0.07 ± 0.05

*24 eyes with previous refractive surgery or cataract surgery were excluded

BCVA, best-corrected visual acuity; SD, standard deviation

### Changes in visual acuity and chorioretinal thickness over 5 years

During the 5-year follow-up, mean logMAR BCVA worsened from 0.20 ± 0.24 to 0.22 ± 0.21 (*P* = 0.023) and axial length increased from 30.80 ± 1.96 mm to 31.17 ± 2.02 mm (*P* < 0.001). Subfoveal CT showed a significant reduction from 67.76 ± 55.39 μm to 58.63 ± 47.95 μm (*P* = 0.005). Foveal RT also decreased significantly from 203.34 ± 35.34 μm to 195.42 ± 41.69 μm (*P* = 0.042). The subfoveal CR ratio also declined over time (from 0.33 ± 0.27 to 0.30 ± 0.24, *P* = 0.036), largely reflecting the relative changes in CT and RT. However, perifoveal CT, RT, and CR ratio did not show statistically significant changes over the same period (Table 2).

**Table 2:** Clinical characteristics at baseline and at 5 Years

	Baseline (mean ± SD)	At 5-year follow-up (mean ± SD)	*P* ^*^
BCVA (logMAR)	0.20 ± 0.24	0.22 ± 0.21	0.023
Axial length (mm)	30.80 ± 1.96	31.17 ± 2.02	< 0.001
Foveal retinal thickness (µm)	203.34 ± 35.34	195.42 ± 41.69	0.042
Perifoveal retinal thickness (µm)	301.19 ± 47.63	300.78 ± 61.64	0.912
Subfoveal choroidal thickness (µm)	67.76 ± 55.39	58.63 ± 47.95	0.005
Perifoveal choroidal thickness (µm)	69.74 ± 65.63	67.23 ± 63.19	0.487
Subfoveal CR ratio	0.33 ± 0.27	0.30 ± 0.24	0.036
Perifoveal CR ratio	0.23 ± 0.22	0.22 ± 0.20	0.264

BCVA, best-corrected visual acuity; CR, choroidal-to-retinal thickness; SD, standard deviation

*P values were derived from GEE (Gaussian family, exchangeable working correlation structure) with patient as the clustering unit. P values reflect the comparison of year 5 to baseline

### Effect of age and axial length on longitudinal chorioretinal thickness changes

**Fig. 3:**
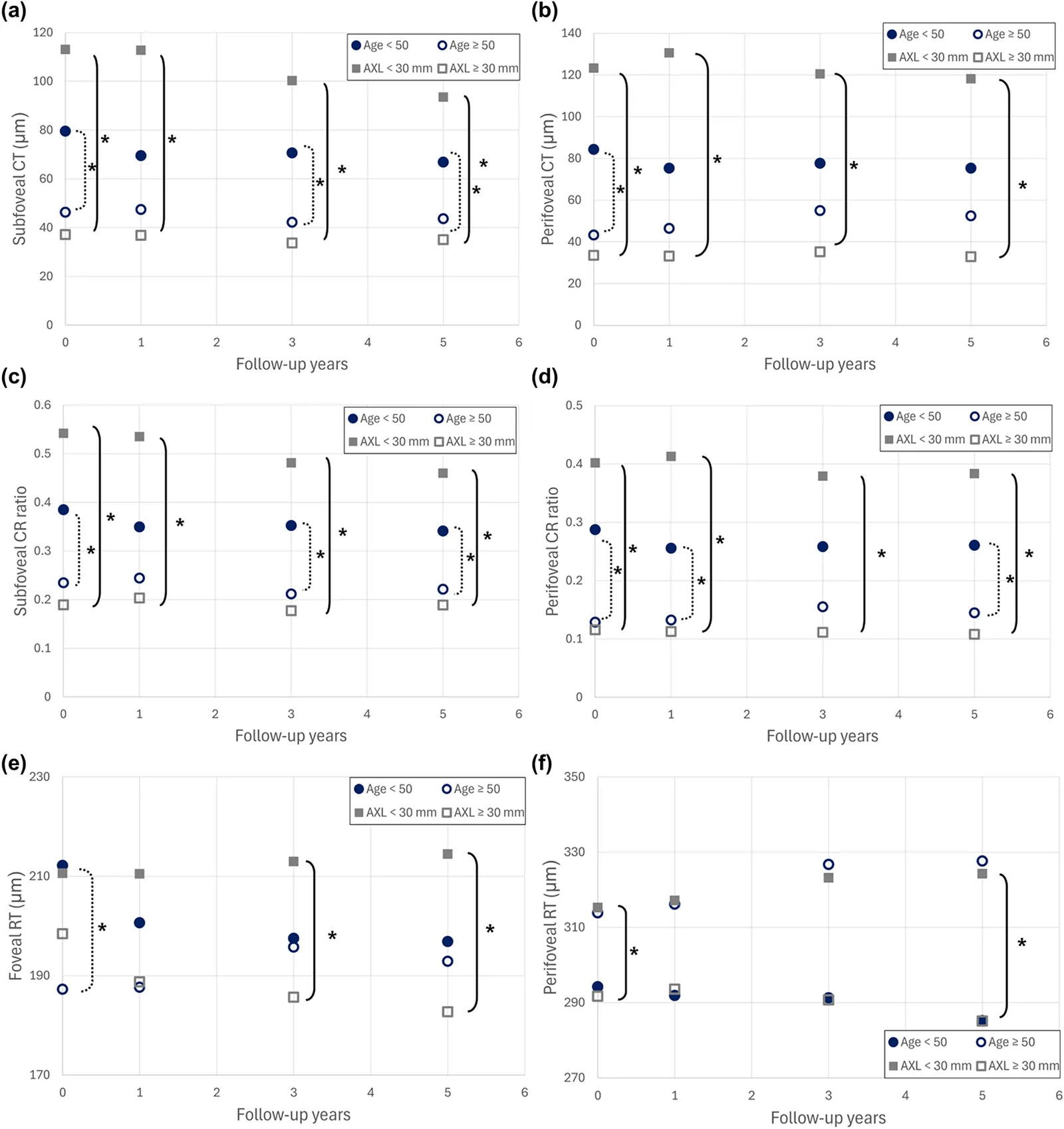
Longitudinal changes in retinal and choroidal thickness over 5 years by age and axial length. (**a**) Subfoveal choroidal thickness (CT), (**b**) perifoveal CT, (**c**) subfoveal choroidal-to-retinal (CR) ratio, (**d**) perifoveal CR ratio, (**e**) foveal retinal thickness (RT), and (**f**) perifoveal RT. Dark blue circles/dotted brackets: age group comparisons; grey squares/solid brackets: axial length group comparisons.* *P* < 0.05

When stratifying by baseline age (< 50 vs. ≥50 years) and axial length (< 30 vs. ≥30 mm), both subfoveal and perifoveal CT and CR ratios remained consistently lower in eyes with longer axial length throughout follow-up (Fig. 3a-d). Younger eyes (< 50 years) showed relatively preserved choroidal structure, with higher subfoveal and perifoveal CT and CR ratios (Fig. 3a-d). Foveal RT tended to be thinner in older and longer eyes, whereas perifoveal RT was thinner in younger eyes with greater elongation (Fig. 3e-f).

### Structural differences and risk factors in rapid vs. moderate axial elongation

**Fig. 4:**
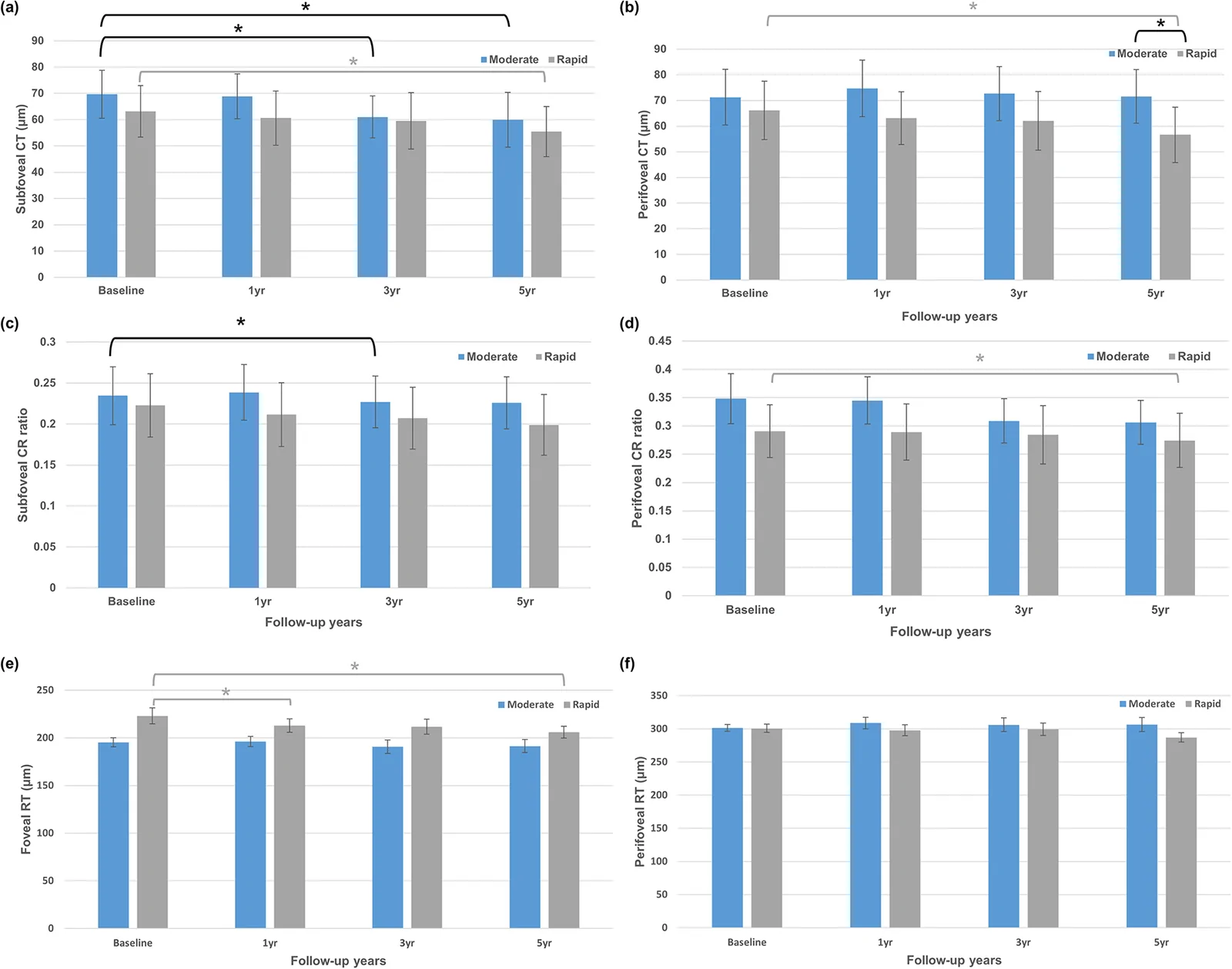
Longitudinal changes in retinal and choroidal thickness over 5 years in moderate and rapid elongation groups. (**a**) Subfoveal choroidal thickness (CT), (**b**) perifoveal CT, (**c**) subfoveal choroidal-to-retinal (CR) ratio, (**d**) perifoveal CR ratio, (**e**) foveal retinal thickness (RT), and (**f**) perifoveal RT. Blue: moderate axial elongation; grey: rapid axial elongation. *P < 0.05

The rapid elongation group was younger (37.8 ± 16.1 vs. 50.0 ± 14.9 years) with greater baseline foveal RT (223.06 vs. 195.28 μm) than the moderate group, though these differences did not reach statistical significance after GEE adjustment (*P* = 0.127 and *P* = 0.056, respectively) (Table 3). Subfoveal and perifoveal CT and CR ratios were consistently lower in the rapid group across all timepoints (Fig. 4a-d), while foveal RT remained thicker throughout follow-up (Fig. 4e). In the moderate group, subfoveal CT and CR ratio declined significantly at years 3 and 5; in the rapid group, significant reductions in perifoveal CT and CR ratio were observed only at year 5. Perifoveal RT showed no significant change in either group (Fig. 4f).

**Table 3:** Clinical characteristics of moderate vs. rapid axial elongation groups

	Total (mean ± SD)	Eyes with rapid annual elongation (mean ± SD)	Eyes with moderate elongation (mean ± SD)	*P* ^*^
Total no. of eyes, *n (%)*	62	18 (29.0)	44 (71.0)	
Age (years)	46.5 ± 16.2	37.8 ± 16.1	50.0 ± 14.9	0.127
Sex, n female (%)	29 (78.4)	10 (71.4)	25 (83.3)	0.198^†^
Baseline axial length (mm)	30.80 ± 1.96	31.22 ± 2.08	30.62 ± 1.91	0.921
Axial elongation rate (mm/year)	0.07 ± 0.05	0.14 ± 0.04	0.05 ± 0.03	< 0.001
Baseline subfoveal CR ratio	0.33 ± 0.27	0.29 ± 0.20	0.34 ± 0.29	0.331
Baseline perifoveal CR ratio	0.23 ± 0.22	0.22 ± 0.16	0.23 ± 0.23	0.367
Baseline subfoveal CT (µm)	67.76 ± 55.39	63.17 ± 41.49	69.64 ± 60.49	0.591
Baseline perifoveal CT (µm)	69.74 ± 65.63	66.11 ± 48.24	71.23 ± 71.99	0.424
Baseline foveal RT (µm)	203.34 ± 35.34	223.06 ± 35.55	195.28 ± 32.29	0.056
Baseline perifoveal RT (µm)	301.19 ± 47.63	300.67 ± 26.41	301.41 ± 54.24	0.648
Baseline posterior staphyloma, n (%)	42 (67.74)	13 (72.22)	29 (65.9)	0.629^†^

CR, choroidal-to-retinal thickness; CT, choroidal thickness; RT, retinal thickness; SD, standard deviation

*P values were derived from GEE (Gaussian family, exchangeable working correlation structure) with patient as the clustering unit

†Fisher’s exact test

In multivariable GEE logistic regression, older age (OR 0.926; 95% CI, 0.880–0.975; *P* = 0.003) and higher subfoveal CR ratio (OR 0.032; 95% CI, 0.003–0.395; *P* = 0.007) were independently associated with a reduced risk of rapid axial elongation, whereas greater baseline foveal RT was associated with an increased risk (OR 1.023; 95% CI, 1.003–1.043; *P* = 0.027) (Table 4).

**Table 4:** Risk factors for rapid axial elongation

Variable	Univariable		Multivariable	
	OR (95% CI)	P*	OR (95% CI)	P*
Age	0.948 (0.915–0.982)	0.003	0.926 (0.880–0.975)	0.003
Sex (female)	0.444 (0.110–1.802)	0.256	—	—
Posterior staphyloma	1.345 (0.367–4.929)	0.655	—	—
Baseline AXL (mm)	1.169 (0.861–1.586*)	0.317	—	—
Subfoveal CT (µm)	0.998 (0.989–1.007)	0.636	Not included^†^	
Perifoveal CT (µm)	0.999 (0.991–1.006)	0.738	Not included^†^	
Foveal RT (µm)	1.025 (1.004–1.046)	0.021	1.023 (1.003–1.043)	0.027
Perifoveal RT (µm)	1.000 (0.990–1.009)	0.944	—	—
Subfoveal CR ratio	0.404 (0.049–3.362)	0.402	0.032 (0.003–0.395)	0.007
Perifoveal CR ratio	0.766 (0.078–7.514)	0.819	Not included^†^	

AXL, axial length; CI, confidence interval; CR, choroidal-to-retinal thickness; CT, choroidal thickness; OR, odds ratio; RT, retinal thickness

*P values were derived from GEE (binomial family, logit link, independence working correlation structure) with patient as the clustering unit

†Excluded due to extreme multicollinearity with subfoveal CR ratio (VIF > 140)

### Univariable and multivariable predictors of posterior staphyloma

In our cohort, 42 eyes (67.7%) exhibited posterior staphyloma at baseline. Compared with eyes without staphyloma, those with staphyloma had significantly longer axial length, thinner subfoveal and perifoveal CT, and lower CR ratios at both locations; retinal thickness did not differ significantly between groups (Additional file 4). In univariable GEE logistic regression, axial length (OR 7.241; 95% CI, 2.166–24.202; *P* = 0.001), subfoveal CR ratio (OR 0.466 per 0.1 unit; 95% CI, 0.268–0.811; *P* = 0.007), and age (OR 1.062; 95% CI, 1.005–1.122; *P* = 0.033) were significantly associated with posterior staphyloma. Complete univariable results are presented in Additional file 5. In the multivariable logistic regression model, axial length was the only significant independent predictor of posterior staphyloma (OR 10.367; 95% CI, 2.055–52.307; *P* = 0.005). Age (OR 1.099; 95% CI, 0.985–1.226, *P* = 0.092) and subfoveal CR ratio (OR 1.274; 95% CI, 0.735–2.210, *P* = 0.388) were not significantly associated with posterior staphyloma (OR 1.099; 95% CI, 0.985–1.226, *P* = 0.092) (Additional file 5). Subfoveal CT, perifoveal CT, and perifoveal CR ratio were excluded from the multivariable model due to multicollinearity with subfoveal CR ratio (variance inflation factor > 10).

## Discussion

In this longitudinal study of eyes with severe high myopia, we characterized long-term structural changes in CT and RT over a 5-year period. To the best of our knowledge, this is the first study to quantify both CT and RT changes in severe myopic eyes without maculopathy across an extended duration. CT was inversely associated with age and axial length, as confirmed by GEE regression analysis (*P* < 0.001, Additional file 6). RT did not show significant associations with either parameter (*P* > 0.05), although it was positively correlated with axial elongation rate (*P* = 0.030, Additional file 6). Notably, foveal RT thinning over 5 years was more apparent in older individuals, contrasting with the findings of paediatric studies in which RT tended to remain stable or increase with age [27]. CR ratio, which integrates both CT and RT into a single metric, also declined with increasing age and axial elongation. However, this trend primarily reflected the more prominent changes observed in CT. Our regression model further confirmed that age, axial length, and axial elongation rate were negative predictors of the subfoveal CR ratio. Taken together, these findings suggest that CT serves as a more sensitive and dynamic structural indicator of myopic progression in this population, whereas CR ratio may be of limited additional value compared to CT alone. These observations are particularly relevant in eyes with extreme axial elongation, where retinal thickness may remain relatively stable despite progressive choroidal atrophy.

During the 5-year follow-up, subfoveal CR ratio was generally higher in eyes with shorter axial lengths (< 30 mm), although no statistically significant differences were noted between age groups. In contrast, perifoveal CR ratio was significantly higher in eyes with axial length < 30 mm and younger age. Both subfoveal and perifoveal CT were significantly thinner in eyes with axial length ≥ 30 mm and age ≥ 50 years, showing greater inter-group separation and progressive decline across time points. In contrast, foveal and perifoveal RT showed relatively stable patterns, with less pronounced group differences and minimal longitudinal changes. These findings underscore the greater vulnerability of the choroid, compared with that of the retina, to age- and axial length–related degeneration in highly myopic eyes.

Interestingly, perifoveal RT was significantly thicker in the older subgroup (age > 50 years), a counterintuitive finding given that foveal RT was thicker in the younger group. One plausible explanation is that the younger group exhibited faster axial elongation (mean 0.086 mm/year vs. 0.053 mm/year, *P* = 0.035), which may lead to earlier or more pronounced perifoveal retinal thinning over time. Similar findings have been reported in longitudinal cohorts, where baseline thinning of the temporal retina was associated with faster axial elongation in both non-myopic and myopic eyes [28]. In contrast, the older group with slower elongation may experience a relative preservation of the perifoveal structure, possibly due to more stabilized or plateaued structural remodelling.

This pattern suggests that selective foveal thinning may precede perifoveal changes, and that younger eyes experiencing rapid axial growth may undergo more diffuse retinal stress. These findings highlight the importance of considering axial elongation rate as a key determinant of regional retinal thickness, beyond age or staphyloma alone. Further longitudinal studies are warranted to clarify whether perifoveal preservation in older adults represents a temporary resilience or a structural plateau after prior thinning.

The rapid elongation group was characterized by younger age and thicker baseline foveal RT. This finding suggests that younger highly myopic eyes may exhibit thicker retinas initially but are prone to more rapid structural remodelling over time. Although CR ratio declined over time in both elongation groups, this change largely mirrored reductions in CT, which was more consistently associated with age, axial length, and elongation rate. Notably, CR ratio was persistently lower in the rapid elongation group, reflecting their disproportionate choroidal thinning.

Multivariable GEE analysis identified axial length and age as significant predictors of both subfoveal and perifoveal CR ratios. In our analysis, lower CR ratio was correlated with worse visual acuity. Given that CR ratio reflects the relative balance between CT and RT, this may indicate that disproportionate choroidal thinning contributes to visual dysfunction in highly myopic eyes. Thus, our findings reinforce the importance of choroidal thickness as a structural marker of visual function and myopia progression in eyes with extreme axial elongation. While the CR ratio may help characterize structural disproportion, it did not appear to be more informative than CT alone in the present study population, warranting further study in larger cohorts with longer follow-up.

The association between CT and age appears to differ across age groups. In paediatric populations aged 4–12 years, CT increases with age, reflecting normal ocular development [29]. Contrastingly, in our older cohort of highly myopic patients (mean age, 46.5 ± 16.2 years), CT showed an age-dependent decline. This finding aligns with previous reports of progressive choroidal thinning associated with both ageing and increasing axial length in myopic eyes [29–31]. In a 2-year longitudinal study of Chinese children, CT showed a marked reduction over time (from 231.03 ± 54.04 μm at baseline to 206.53 ± 59.71 μm), while RT remained relatively stable (from 251.12 ± 15.91 μm to 253.47 ± 15.74 μm) [32]. This difference may be attributed to the distinct structural and physiological characteristics of the choroid and retina. The choroid, as a vascular tissue, exhibits greater physiological plasticity and susceptibility to remodelling due to its dynamic blood flow and volume regulation, whereas the retina has a more stable structure and a less variable blood supply [33].

The relatively high standard deviations observed in subfoveal and perifoveal CT measurements in our cohort likely reflect inter-individual variability in choroidal morphology among eyes with severe high myopia (mean axial length 30.8 mm) [34, 35]. This variability may be attributed to a combination of pronounced choroidal thinning and anatomical differences such as posterior staphyloma configuration, local scleral curvature, and choroidal vascular remodelling [36–38]. Particularly, the large variance in perifoveal CT may partly explain the lack of significant longitudinal change in that region, as well as the nonsignificant difference in perifoveal CR ratio. However, because our imaging was limited to standard field OCT, detailed topographic characterization of staphyloma morphology was not feasible. These limitations highlight the need to consider local anatomical differences when analysing choroidal thickness in pathologic myopia.

The structural differences observed between eyes with and without staphyloma, particularly the more severe choroidal atrophy reflected by lower CT and CR ratios, support the concept that staphyloma development is more closely associated with progressive axial elongation than with ageing itself. Although eyes with staphyloma tended to be older, age was not independently associated with staphyloma after accounting for inter-eye correlation, whereas axial length remained a significant predictor in multivariable analysis. In contrast, retinal thickness remained relatively preserved despite advanced structural elongation, suggesting that choroidal alterations may precede substantial retinal structural changes during staphyloma formation. The strong association with axial length is consistent with previous reports in highly myopic eyes, though the magnitude of the OR may reflect the more severely myopic spectrum of our cohort (mean axial length 30.80 mm) compared with prior studies [39]. This difference may be attributed to the greater severity of myopia in our cohort reflecting the increased biomechanical stress due to severe axial elongation. Moreover, because CT and RT were measured specifically at the subfoveal and perifoveal regions, staphylomas that did not involve these areas, such as the inferior or peripapillary types, may have limited impact on the measured parameters. In fact, 95.2% of staphyloma cases in our study did not show a staphyloma edge within 1,000 μm of the foveal centre. As shown in Additional file 7, the CTs at these locations were relatively thicker than typically observed at the staphyloma margin, where choroidal thinning is most pronounced [36]. Therefore, the lack of significant association between staphyloma and CR ratio may reflect these measurement limitations. Overall, our results suggest that choroidal and retinal measurements in the absence of ultra-widefield OCT imaging may not fully capture the structural severity of posterior staphyloma, particularly.

Notably, the pattern of disproportionate choroidal thinning in our cohort parallels age-related changes, where CT decreases significantly while RT remains stable [40–43]. This suggests that choroidal atrophy may be an early pathologic feature in myopic eyes, potentially preceding axial elongation and staphyloma formation. Future research investigating structural markers and their potential responsiveness to therapeutic interventions targeting choroidal remodelling may also offer insight into modifying the course of myopia.

Several limitations of this study should be acknowledged. First, the relatively small sample size may have limited the statistical power and generalizability of the findings. Second, axial length was measured using the IOL Master 500 through 2018 and the IOL Master 700 thereafter; although both devices demonstrate high agreement, this transition may have introduced minor measurement variability. Third, the exclusion of eyes with pathologic myopia limits the generalizability of our findings to the full spectrum of myopic maculopathy. Fourth, the absence of a control group with emmetropic or low myopia is another limitation. Previous studies have shown that emmetropic individuals typically exhibit a CR ratio greater than 1.0 [44], whereas our pathologic myopia cohort demonstrated uniformly low CR ratios with relatively small RT variation compared to CT. This narrow range may have limited the utility of the CR ratio in our population. Future studies including a broader refractive spectrum and stratifying patients by CR ratio thresholds may better elucidate the relationship between choroidal-retinal thickness patterns and clinical outcomes. While CR ratio may aid in characterizing the relative contributions of CT and RT, its role as a prognostic biomarker warrants further validation in larger and more diverse populations.

## Conclusions

In eyes with severe high myopia, both subfoveal CT and CR ratio were significantly associated with age and axial length. Over 5 years, subfoveal CT, foveal RT, and subfoveal CR ratio all decreased significantly, with CT showing the most consistent structural change in relation to age and axial length. Importantly, after GEE adjustment, a higher subfoveal CR ratio independently predicted a lower risk of rapid axial elongation, suggesting that the CR ratio may capture a distinct dimension of structural vulnerability beyond CT alone. Together, these findings support subfoveal CT as a sensitive structural marker of myopic progression and the subfoveal CR ratio as a potential prognostic biomarker for axial elongation in severe high myopia.

## Supplementary Information

Below is the link to the electronic supplementary material.

Supplementary Material 1: Additional file 1. Format: PNG. Title: Retinal and choroidal thickness in relation to refractive error. Scatter plots of (a) choroidal thickness, (b) choroidal-to-retinal thickness ratio, and (c) retinal thickness. Subfoveal/foveal values (green); perifoveal values (blue). CR ratio, choroidal-to-retinal thickness ratio; CT, choroidal thickness; RT, retinal thickness. 24 eyes with previous refractive surgery or cataract surgery were excluded. Description: Scatter plots of choroidal thickness, choroidal-to-retinal thickness ratio, and retinal thickness in relation to baseline refractive error.

Supplementary Material 2: Additional file 2. Format: PNG. Title: Visual acuity in relation to retinal and choroidal thickness. Scatter plots showing baseline best-corrected visual acuity in relation to choroidal thickness (a), choroidal-to-retinal thickness ratio (b), and retinal thickness (c). Subfoveal/foveal values (green); perifoveal values (blue). CR ratio, choroidal-to-retinal thickness ratio; CT, choroidal thickness; RT, retinal thickness.. Description: Scatter plots of choroidal thickness, choroidal-to-retinal thickness ratio, and retinal thickness in relation to best-corrected visual acuity.

Supplementary Material 3: Additional file 3. Format: DOC. Title: Multivariable Regression for Subfoveal and Perifoveal Choroidal-to-Retinal Thickness Ratio. Description: A table showing the results of a multivariable regression analysis of subfoveal and perifoveal CR ratios.

Supplementary Material 4: Additional file 4. Format: DOC. Title: Clinical Features Based on Posterior Staphyloma. Description: Comparison of clinical features between eyes with and without posterior staphyloma.

Supplementary Material 5: Additional file 5. Format: DOC. Title: Predictors of Posterior Staphyloma. Description: Multivariable logistic regression analysis of risk factors for posterior staphyloma.

Supplementary Material 6: Additional file 6. Format: DOC. Title: Multivariable Regression Analysis of Subfoveal Choroidal Thickness and Foveal Retinal Thickness. Description: Regression coefficients from multivariable analyses with subfoveal choroidal thickness and foveal retinal thickness as dependent variables.

Supplementary Material 7: Additional file 7. Format: TIF. Title: Optical coherence tomography image from a representative patient with posterior staphyloma. The subfoveal and perifoveal choroid (within 1,000 μm) appears thicker (white arrowheads) than at the edge of the staphyloma (yellow arrowheads). Description: This image shows that subfoveal and perifoveal choroidal thickness is greater than that at the posterior staphyloma edge.

## Data Availability

The datasets used and/or analysed during the current study are available from the corresponding author on reasonable request.

## Abbreviations

BCVA: Best-corrected visual acuity
CR: Choroidal-to-retinal thickness
CT: Choroidal thickness
RT: Retinal thickness
OCT: Optical coherence tomography
