# Supplementary material for: Five-year structural changes in severe high myopia: the role of choroidal and retinal thickness
Source: BMC Ophthalmol. 2026 Jul 28;26:672. doi: 10.1186/s12886-026-05076-1 (PMC13693147; doi:10.1186/s12886-026-05076-1)
Supplement: Supplementary file 1 [file 12886_2026_5076_MOESM3_ESM.docx]

**Additional file 3**. Multivariable Regression for Subfoveal and Perifoveal Choroidal-to-Retinal Thickness Ratio

|  | **Subfoveal CR ratio** | | **Perifoveal CR ratio** | |
| --- | --- | --- | --- | --- |
| **Independent variable** | **Coefficients** | ***P^*^*** | **Coefficients** | ***P^*^*** |
| **Age** | -0.007 | < 0.001 | -0.006 | < 0.001 |
| **Axial length** | -0.091 | < 0.001 | -0.075 | < 0.001 |
| **Axial elongation rate** | -1.077 | 0.018 | -0.557 | 0.082 |
| **Sex** | -0.050 | 0.197 | -0.071 | 0.047 |

CR, choroidal-to-retinal thickness ratio.

*All P values were derived from generalized estimating equations (GEE; Gaussian family, exchangeable working correlation structure) with patient as the clustering unit to account for inter-eye correlation.
