# Supplementary material for: Five-year structural changes in severe high myopia: the role of choroidal and retinal thickness
Source: BMC Ophthalmol. 2026 Jul 28;26:672. doi: 10.1186/s12886-026-05076-1 (PMC13693147; doi:10.1186/s12886-026-05076-1)
Supplement: Supplementary file 2 [file 12886_2026_5076_MOESM4_ESM.docx]

**Additional file 4**. Clinical Features Based on Posterior Staphyloma

| **Characteristic** | **Posterior Staphyloma (n=42)**  **(mean ± SD)** | **No posterior staphyloma (n=20)**  **(mean ± SD)** | ***P* (t-test)^*^** |
| --- | --- | --- | --- |
| **Age (years)** | 50.74 ± 13.03 | 37.55 ± 17.79 | 0.456 |
| **Axial length (μm)** | 31.60 ± 1.78 | 29.01 ± 0.73 | < 0.001 |
| **Axial elongation rate (μm/year)** | 0.07 ± 0.05 | 0.07 ± 0.05 | 0.582 |
| **Sex (n female) (%)** | 30 (71.4%) | 18 (90.0%) | 0.192^†^ |
| **Subfoveal CT (μm)** | 45.98 ± 36.21 | 113.50 ± 59.29 | 0.002 |
| **Perifoveal CT (μm)** | 43.48 ± 38.79 | 124.90 ± 74.08 | 0.001 |
| **Foveal RT (μm)** | 204.67 ± 39.36 | 200.55 ± 23.34 | 0.757 |
| **Perifoveal RT (μm)** | 297.90 ± 52.95 | 308.10 ± 31.02 | 0.166 |
| **Subfoveal CR** | 0.22 ± 0.16 | 0.57 ± 0.29 | < 0.001 |
| **Perifoveal CR** | 0.14 ± 0.12 | 0.41 ± 0.24 | 0.001 |

CR, choroidal-to-retinal thickness ratio; CT, choroidal thickness; RT, retinal thickness, SD, standard deviation.

**P* values derived from generalized estimating equations (GEE; Gaussian family, exchangeable working correlation structure) with patient as the clustering unit. **P* < 0.05; ** *P* < 0.01; *** *P* < 0.001.

^†^Fisher's exact test.
