# Supplementary material for: Five-year structural changes in severe high myopia: the role of choroidal and retinal thickness
Source: BMC Ophthalmol. 2026 Jul 28;26:672. doi: 10.1186/s12886-026-05076-1 (PMC13693147; doi:10.1186/s12886-026-05076-1)
Supplement: Supplementary file 3 [file 12886_2026_5076_MOESM5_ESM.docx]

**Additional file 5.** Predictors of Posterior Staphyloma

| **Variable** | **Univariable** | | **Multivariable** | |
| --- | --- | --- | --- | --- |
|  | **OR (95% CI)** | ***P**** | **OR (95% CI)** | ***P**** |
| **Age (years)** | 1.062 (1.005, 1.122) | 0.033 | 1.099 (0.985, 1.226) | 0.092 |
| **Sex (female)** | 0.278 (0.030, 2.573) | 0.259 | — | — |
| **Baseline AXL (mm)** | 7.241 (2.166, 24.202) | 0.001 | 10.367 (2.055, 52.307) | 0.005 |
| **Elongation rate** | 0.994 (0.287, 3.445) | 0.992 | — | — |
| **Subfoveal CT (μm)^‡^** | 0.737 (0.584, 0.930) | 0.010 | Not included^†^ | — |
| **Perifoveal CT (μm)^‡^** | 0.773 (0.667, 0.896) | <0.001 | Not included^†^ | — |
| **Foveal RT (μm)^‡^** | 1.035 (0.885, 1.210) | 0.669 | — | — |
| **Perifoveal RT (μm)^‡^** | 0.957 (0.847, 1.081) | 0.478 | — | — |
| **Subfoveal CR ratio^§^** | 0.466 (0.268, 0.811) | 0.007 | 1.274 (0.735, 2.210) | 0.388 |
| **Perifoveal CR ratio^§^** | 0.458 (0.282, 0.742) | 0.002 | Not included^†^ | — |

AXL, axial length; CI, confidence interval; CR, choroidal-to-retinal thickness; CT, choroidal thickness; OR, odds ratio; RT, retinal thickness.

**P* values were derived from generalized estimating equations (GEE; binomial family, logit link, independence working correlation structure) with patient as the clustering unit.

†Excluded from multivariable model due to multicollinearity with subfoveal CR ratio (VIF > 10).

‡OR expressed per 10 μm increase.

§OR expressed per 0.1 unit increase.
