# Supplementary material for: Five-year structural changes in severe high myopia: the role of choroidal and retinal thickness
Source: BMC Ophthalmol. 2026 Jul 28;26:672. doi: 10.1186/s12886-026-05076-1 (PMC13693147; doi:10.1186/s12886-026-05076-1)
Supplement: Supplementary file 4 [file 12886_2026_5076_MOESM6_ESM.docx]

**Additional file 6**. Multivariable Regression Analysis of Subfoveal Choroidal Thickness and Foveal Retinal Thickness.

|  | **Subfoveal choroidal thickness** | | **Foveal retinal thickness** | |
| --- | --- | --- | --- | --- |
| **Independent variable** | **Coefficients** | ***P^*^*** | **Coefficients** | ***P^*^*** |
| **Age** | -1.398 | <0.001 | -0.166 | 0.601 |
| **Axial length** | -19.615 | <0.001 | -1.425 | 0.503 |
| **Axial elongation rate** | -133.755 | 0.160 | 256.732 | 0.030 |
| **Sex** | -13.973 | 0.075 | -9.284 | 0.409 |

*All P values were derived from generalized estimating equations (GEE; Gaussian family, exchangeable working correlation structure) with patient as the clustering unit to account for inter-eye correlation.
